# Multi-order graph attention network for water solubility prediction and interpretation

**DOI:** 10.1038/s41598-022-25701-5

**Published:** 2023-03-02

**Authors:** Sangho Lee, Hyunwoo Park, Chihyeon Choi, Wonjoon Kim, Ki Kang Kim, Young-Kyu Han, Joohoon Kang, Chang-Jong Kang, Youngdoo Son

**Affiliations:** 1 Department of Industrial and Systems Engineering, Dongguk University-Seoul, Seoul, 04620 South Korea; 2 Data Science Laboratory (DSLAB), Dongguk University-Seoul, Seoul, 04620 South Korea; 3 Division of Future Convergence (HCI Science Major), Dongduk Women’s University, Seoul, 02748 South Korea; 4 Department of Energy Science, Sungkyunkwan University (SKKU), Suwon, 16419 South Korea; 5 Center for Integrated Nanostructure Physics (CINAP), Institute for Basic Science (IBS), Sungkyunkwan University (SKKU), Suwon, 16419 South Korea; 6 Department of Energy and Materials Engineering, Dongguk University-Seoul, Seoul, 04620 South Korea; 7 School of Advanced Materials Science and Engineering, Sungkyunkwan University (SKKU), Suwon, 16419 South Korea; 8 KIST-SKKU Carbon-Neutral Research Center, Sungkyunkwan University (SKKU), Suwon, 16419 South Korea; 9 Department of Physics, Chungnam National University, Daejeon, 34134 South Korea

**Keywords:** Cheminformatics, Machine learning, Computer science, Information technology

## Abstract

The water solubility of molecules is one of the most important properties in various chemical and medical research fields. Recently, machine learning-based methods for predicting molecular properties, including water solubility, have been extensively studied due to the advantage of effectively reducing computational costs. Although machine learning-based methods have made significant advances in predictive performance, the existing methods were still lacking in interpreting the predicted results. Therefore, we propose a novel multi-order graph attention network (MoGAT) for water solubility prediction to improve the predictive performance and interpret the predicted results. We extracted graph embeddings in every node embedding layer to consider the information of diverse neighboring orders and merged them by attention mechanism to generate a final graph embedding. MoGAT can provide the atomic-specific importance scores of a molecule that indicate which atoms significantly influence the prediction so that it can interpret the predicted results chemically. It also improves prediction performance because the graph representations of all neighboring orders, which contain diverse range of information, are employed for the final prediction. Through extensive experiments, we demonstrated that MoGAT showed better performance than the state-of-the-art methods, and the predicted results were consistent with well-known chemical knowledge.

## Introduction

Since most chemical and biological reactions occur when dissolved in water, the water solubility of a molecule or polymer is an important factor in various academic and industrial fields such as chemistry, biochemistry, food engineering, medical, and pharmaceutical industries. For example, biological activities such as the reaction between proteins^1^, protein and nucleic acid structures^2^, protein-substrate binding^3^, and protein folding^3^ are conducted in the liquid state^4^; thus, solubility plays an important role in dosage forms and desired concentration of drugs to achieve the required pharmacological response^5^.

Accurate measurement of the water solubility of a molecule involves rigorous and time-consuming experiments that are highly sensitive to the external environment. Furthermore, although there are several theoretical models for computing solubility^6,7^, these models were empirically constructed using only a small amount of experimental data. Therefore, building a general empirical model is challenging for a large set of experimental data using the existing theoretical models.

Recently, to overcome this limitation, various machine learning (ML)-based methods have been widely introduced to predict solubility and other molecular properties using molecular features, including molecular weights, ring structures, and aromatic properties^8–11^. Some studies have improved the prediction performance with graphs consisting of nodes and edges representing atoms and bonds, respectively, as inputs^12–15^. To effectively capture the structural characteristics of the constructed graphs, they used various graph neural network (GNN)-based methods, including message passing neural network (MPNN)^13^ and its variants with attention^16,17^, to predict molecular property. They also demonstrated the graph could effectively represent the structural characteristics of the molecules. Although the GNN-based methods improved the predictive performance of molecular properties, most of them failed to interpret factors that substantially impact molecular properties prediction^18^. Analyzing the impacts of each factor in molecules provides confidence in the results; thus, the GNN-based methods should interpret which atoms in a molecule highly affect the prediction in a similar way to the well-known chemical knowledge. AttentiveFP^14^, one of the GNN-based chemical property prediction methods, can provide the importance of each atom in predicting molecular properties. Specifically, AttentiveFP well-interpreted the predicted results through the importance of each atom obtained from neighbors’ information of the final node embedding layer, as well as achieved state-of-the-art performance. However, it can only consider the neighbors’ information from the last node embedding layer and cannot directly reflect the information of the different neighboring orders obtained from the other layers.

Therefore, we propose a multi-order graph attention network (MoGAT) for water solubility prediction to improve the performances of prediction and interpretation with diverse aspects of neighbors’ information. First, for each node embedding layer, we derived node embeddings, which imply the hidden states of each atom, updated by reflecting information of its neighbors. Then, graph embeddings representing the whole molecule at every node embedding layer are calculated. Finally, a final graph embedding is derived by giving weights calculated with the softmax function to the graph embeddings. The graph embeddings obtained from every node embedding layer reflect the information of different neighboring orders; thus, the final graph embedding provides useful information in predicting water solubility. In addition, the weights calculated with the softmax function to the graph embeddings imply the importance scores of each atom so that we can interpret the effect of each atom on the predicted results.

To verify the predictive performance of MoGAT, we performed several experiments with extensive datasets. As a result, we demonstrated that MoGAT achieved better performance than the existing GNN-based methods. Furthermore, we interpreted which atoms in a molecule are important for water solubility by deriving atomic-specific importance by integrating information of diverse neighboring orders. The importance scores of atoms were also consistent with the chemical intuitions from the existing calculation results^19^.

The rest of this paper is organized as follows. In the next section, the preliminaries and a detailed algorithm of the proposed method are described. Then, we present the experimental results on various benchmark datasets, which demonstrate the effectiveness of MoGAT. Finally, we conclude with a discussion on the limitations of MoGAT and mention of future research directions.

## Methodology

In this section, we first briefly explain the attention mechanism applied to GNNs. Then, we propose a novel graph attention network, MoGAT, for predicting and interpreting water solubility.

### Graph neural networks with attention mechanism

In general, GNNs used to predict molecular property consist of two phases: a message-passing phase between nodes and a readout phase. The message-passing phase repeatedly updates the hidden state of each node by reflecting information from its neighboring nodes. In the readout phase, a graph embedding is derived by unifying the hidden states of all nodes that have been updated in the message passing phase.

The attention mechanism^20^ can present the importance of each input variable related to a target value. Bahdanau et al.^20^ first introduced the attention mechanism for the machine translation task. However, it has recently been employed in various tasks, such as image processing^21^, speech recognition^22^, and graph analysis^23^, owing to its advantage that it can enable models to focus on certain important information. When applying the attention mechanism to the GNNs, using the regional information around the target node is crucial. To calculate the importance score of each node, we first concatenate hidden states of a node *v* to be updated and a neighboring node *n*. Then, as the following Eq. (1), we generate $e_{vn}$ by linearly transforming the concatenated vector with a learnable parameter matrix *W* and applying the leaky rectified linear unit (LeakyReLU) as a non-linear activation function.

1 $$\begin{aligned} e_{vn}=\texttt {LeakyReLU}(W\cdot [h_v;h_n])= {\left\{ \begin{array}{ll} W\cdot [h_v;h_n], &{} \text {if } W\cdot [h_v;h_n] \ge 0, \\ 0.01 \times W\cdot [h_v;h_n],&{} \text {otherwise}, \end{array}\right. } \end{aligned}$$

where $h_v$ and $h_n$ denote hidden states of the nodes, *v* and *n*, respectively. Second, an importance attention score $s_{vn}$ for *v* of *n* is obtained by normalizing $e_{vn}$ for all neighbor nodes of *v* using the softmax function as follows:

2 $$\begin{aligned} s_{vn}=softmax(e_{vn})=\frac{exp(e_{vn})}{\sum _{n \in \mathcal {N}(v)}exp(e_{vn})}, \end{aligned}$$

where $\mathcal {N}(v)$ denotes all neighbor nodes of *v*. Next, as in Eq. (3), the context vector $c_v$ consisting of the importance scores for the node *v* is calculated by linearly transforming $h_n$ with the learnable parameter matrix *V*, taking a weighted sum of it using $s_{vn}$ as weights, and applying the exponential linear unit (ELU) as a non-linear activation function.

3 $$\begin{aligned} c_v= \texttt {ELU}\left(\sum _{n \in \mathcal {N}(v)} s_{vn} \cdot V \cdot h_n \right)= {\left\{ \begin{array}{ll} \sum _{n \in \mathcal {N}(v)} s_{vn} \cdot V \cdot h_n, &{} \text {if } \sum _{n \in \mathcal {N}(v)}s_{vn} \cdot V \cdot h_n > 0, \\ exp(\sum _{n \in \mathcal {N}(v)}s_{vn} \cdot V \cdot h_n)-1, &{} \text {if } \sum _{n \in \mathcal {N}(v)}s_{vn} \cdot V \cdot h_n \le 0. \end{array}\right. } \end{aligned}$$

Finally, for updating $h_v$, one of the recurrent neural networks, such as the gated recurrent unit (GRU)^24^ and long short-term memory^25^, is used to generate messages among nodes.

### MoGAT: multi-order graph attention network

**Figure 1:**
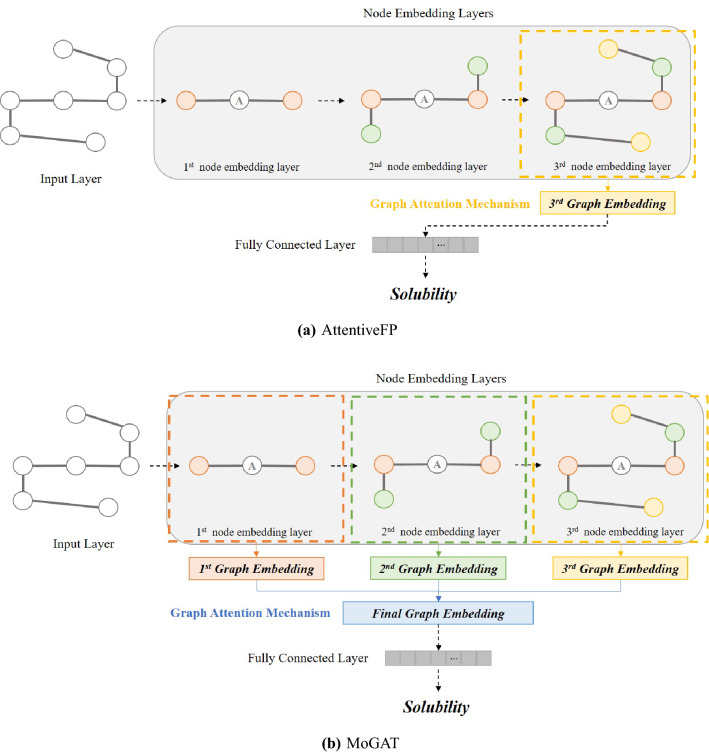
Overviews of (**a**) AttentiveFP and (**b**) MoGAT. The first, second, and third-order neighboring nodes are colored red, green, and yellow, respectively. In AttentiveFP, a graph embedding is obtained only from the final (third) node embedding layer. In contrast, MoGAT constructs graph embeddings corresponding to all node embedding layers, and the graph embeddings are combined into one final graph embedding (blue) via the attention mechanism.

Although AttentiveFP achieved good predictive performance and presented important atoms affecting the final prediction, it cannot directly reflect information of diverse neighboring orders since it computes importance scores at the final node embedding layer only, as shown in Fig. 1a. Note that the range of information (neighboring orders) of nodes reflected in the hidden representations increases as the message passing phase is repeated. For example, a target node receives information from the neighboring nodes directly connected to the target node. Then, in the next update, the information for the neighbors of the nodes adjacent to the target node is also delivered to the target node because the adjacent nodes were updated by reflecting their neighbors’ information in the previous step. Thus, for each update of node embedding, information of one additional neighboring order is included.

Therefore, we propose MoGAT that directly reflects the information of diverse neighboring orders from each node embedding layer to the final prediction. The architecture of the proposed MoGAT is shown in Fig. 1b. Specifically, we first generate graph embeddings in all node embedding layers. Then, we calculate the importance scores of each graph embedding and construct a final graph embedding using the calculated importance scores as weights of corresponding graph embeddings. In other words, compared with AttentiveFP, we used the attention mechanism (Eqs. (1)–(3)) to derive the final graph embedding as well as those of each node embedding layer. Thus, we can improve prediction and interpretation performances by obtaining useful representation and importance scores with richer neighbors’ information.

For obtaining a graph representation (graph embedding) of a node embedding layer, we first define a virtual super node $c_{super}$, which is assumed to be connected to all nodes (atoms). The graph representation of $c_{super}$ is calculated same procedures as in Eqs. (1)–(3). Then, we adopt GRU to update $h_{super}$, the hidden state of $c_{super}$, that equals the graph embedding of the node embedding layer. The GRU can efficiently capture the messages when updating the hidden state of the node by using update and reset gates at each update step^26,27^; hence, the GRU has been used in many previous studies in the chemistry domain^28,29^. Given the previous hidden state, $h^{i-1}_v$, and context vector, $c^{i-1}_v$, of the node *v*, the hidden state is updated as follows:

4 $$\begin{aligned} r^i_v = \sigma \bigg(W_r \cdot [h^{i-1}_v; c^i_v]\bigg), \end{aligned}$$

5 $$\begin{aligned} z^i_v = \sigma \bigg(W_z \cdot [h^{i-1}_v; c^i_v]\bigg), \end{aligned}$$

6 $$\begin{aligned} h'^i_v = \texttt {tanh}\bigg(W_h \cdot [r^i_v \circ h^{i-1}_v; c^i_v]\bigg), \end{aligned}$$

7 $$\begin{aligned} h^i_v = (1-z^i_v) \circ h'^i_v + z^i_v \circ h^{i-1}_v, \end{aligned}$$

where $r^i_v$ and $z^i_v$ are the states of the reset and update gates, respectively, $W_r$, $W_z$, and $W_h$ are the learnable parameters, $\sigma$ is a sigmoid function, and $\circ$ is the Hadamard product. Subsequently, we calculate the weights of each atom for the graph embedding using the attention mechanism as in Eq. (8).

8 $$\begin{aligned} Attention(\mathcal {G})=softmax\bigg(\frac{\mathcal {G}\mathcal {G}^T}{\sqrt{d_\mathcal {G}}}\bigg), \end{aligned}$$

where $\mathcal {G}$ is a set in which graph embeddings are concatenated, and $d_\mathcal {G}$ denotes the dimension (finger print) of $\mathcal {G}$. Next, a final graph embedding is derived by the dot-product between the weights and corresponding graph embeddings. Finally, we use a one-layered fully connected network (FC) to predict the target, water solubility, with the final graph embedding.

Thus, MoGAT can track how each atom in the molecule affected the final graph embedding because all intermediate graph embeddings were generated by weighting the node embeddings of all atoms with the derived attention scores. In other words, our method can interpret the importance of each atom for the molecule for the property prediction through the size of attention scores.

To summarize, the proposed method first creates node and graph embeddings for each neighboring order in the message-passing phase. Then, the graph attention mechanism is used to update the node embeddings and generate the graph embeddings obtained by the virtual super node connected to all atoms for each neighboring order. Next, the final graph embedding is obtained by the weighted sum of the graph embeddings, where the weights of each graph embedding are calculated using the attention mechanism, as above Eq. (8), in the readout phase. Finally, the final prediction result is computed with the obtained final graph embedding using the FC. We summarized the overall procedures of the proposed MoGAT in Algorithm 1.

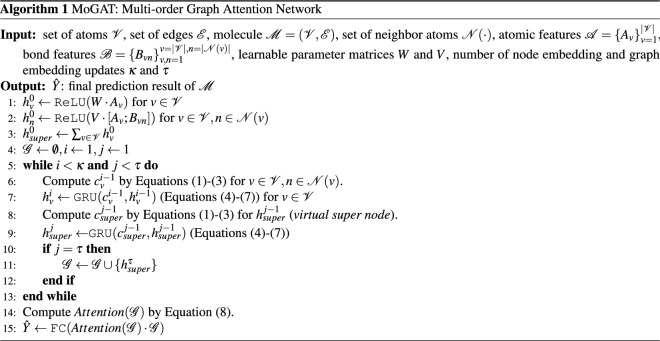



## Experiments

To verify the performance for prediction and interpretation of MoGAT, we used a public water solubility dataset, *Estimated Solubility* (ESOL^11^), to evaluate the predictive performance of MoGAT. ESOL contains experimentally measured solubility values of 1,128 molecular compounds with an average of − 3.05 and a standard deviation of 2.1 where the unit is *log solubility in mols per liter* (logS).

In addition, we compared MoGAT with several baseline models for predicting molecular properties based on GNNs, such as graph convolution neutral network (GCN)^30^, Weave^12^, MPNN^13^, and AttentiveFP^14^. GCN transfers and receives information through a weight matrix of neighboring nodes. For Weave and MPNN, additional edge features and node features are used when the information is transferred to neighboring nodes. Moreover, MPNN reflects the connectivity characteristics between atoms by integrating information from the edge features and node features for the enhancement of performance. AttentiveFP is an interpretable GNN based on MPNN. It constructs a graph representation by applying the attention mechanism at both atomic and molecular levels. The baseline models were implemented using the codes uploaded on the GitHub^31^.

Training, validation, and test datasets were randomly separated by 80%, 10%, and 10% of the total number of data, respectively. We set the hyperparameters, such as $\ell _2$ weight decay and training epoch, equal to Xiong et al.^14^ for fair comparison. Additional hyperparameters for MoGAT, including the number of node embedding and graph embedding updates, are found by the random search. The search ranges and selected optimal hyperparameters for MoGAT are listed in Table 1.

**Table 1:** Search ranges for hyperparameters of MoGAT.

Hyperparameter	Search range
$\kappa$	1, **2**, 3, 4, 5, 6
$\tau$	1, **2**, 3, 4, 5, 6
$d_\mathcal {G}$	100, 150, **200**, 250, 300, 350, 400
Dropout rate	0.1, **0.2**, 0.3, 0.4, 0.5
Learning rate	0.1, **0.01**, 0.001, 0.0001

The optimal values are highlighted in boldface. $\kappa$ and $\tau$ denote the number of node embedding and graph embedding updates, respectively. In addition, $d_\mathcal {G}$ denotes dimension of the embeddings.

For a fair comparison, we used input features for all experiments as same in Xiong et al.^14^ and listed them in Table 2.

**Table 2:** List of input features.

	Features	Size	Type	Description
Atomic	Atom symbol	16	One-hot	B, C, N, O, F, Si, P, S, Cl, As, Se, Br, Te, I, At, metal
	Degree	6	One-hot	Number of covalent bonds
	Formal charge	1	Integer	Electrical charge
	Radical electrons	1	Integer	Number of radical electrons
	Hybridization	6	One-hot	sp, sp2, sp3, sp3d, sp3d2, other
	Aromaticity	1	Binary	Ehether the atom is part of an aromatic system
	Hydrogens	5	One-hot	Number of connected hydrogens
	Chirality	1	Binary	Whether the atom is chiral center
	Chirality type	2	One-hot	R, S
Bond	Bond type	4	One-hot	single, double, triple, aromatic
	Conjugation	1	Binary	Whether the bond is conjugated
	Ring	1	Binary	Whether the bond is in ring
	Stereo	4	One-hot	StereoNone, StereoAny, StereoZ, StereoE

For all experiments, we repeated the separation of the dataset five times and reported the averaged results to reduce the effect of randomness.

### Experimental results

**Figure 2:**
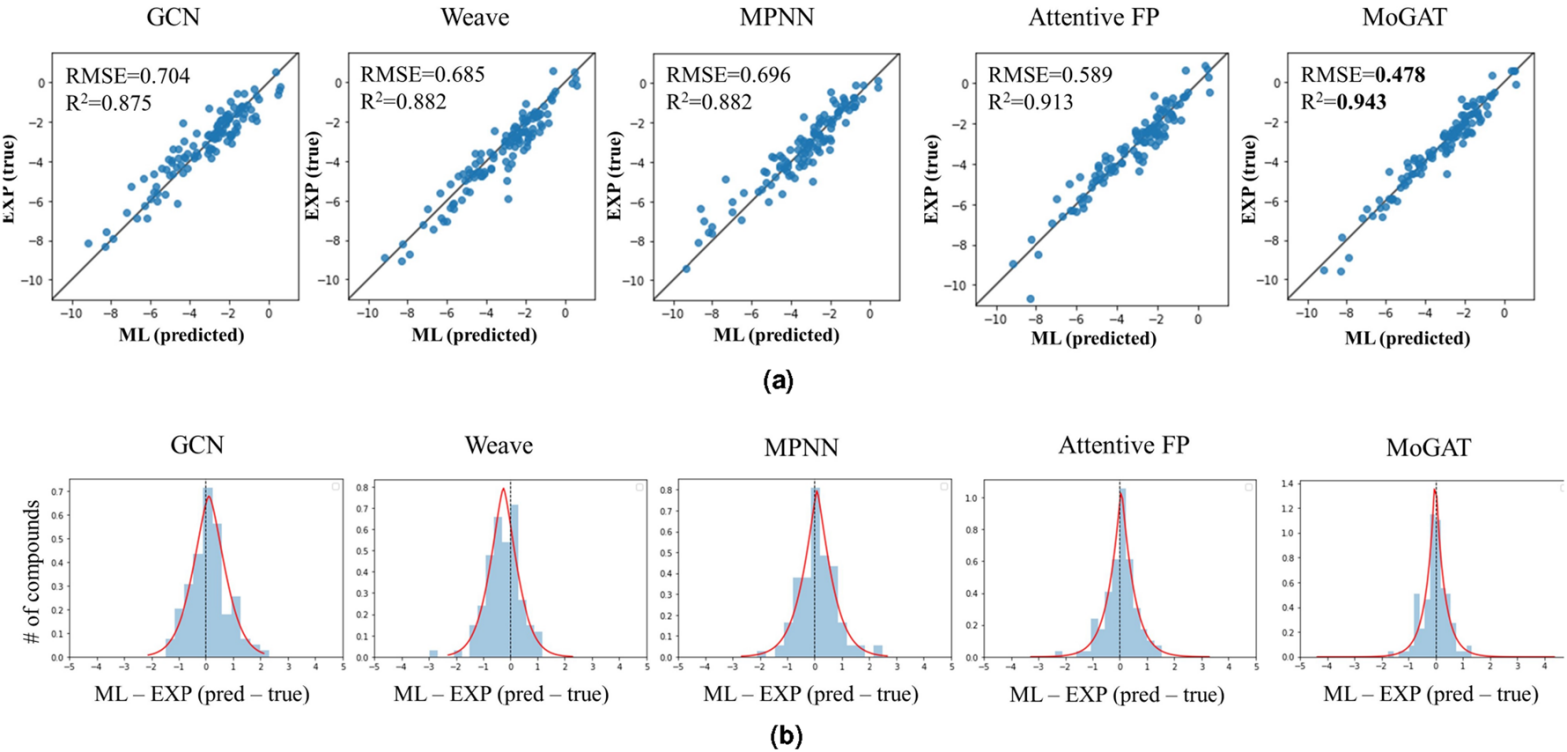
Comparison of the proposed method and other baseline models in estimating water solubility. (**a**) Scatter plots of predicted and experimental solubility values, where the x-axis and y-axis indicate the predicted and experimental solubility values. ML and EXP indicate the predicted and experimental solubility values, respecitvely. (**b**) Generalized error distribution of the errors between ML and EXP. The unit of water solubility is logS.

Figure 2a shows root mean squared error (RMSE) and R-squared ($R^2$) of each method for estimating water solubility. The points derived by MoGAT located closer to a diagonal line ($y=x$) than the points by the other baseline methods, which implies that MoGAT predicts the water solubility of the molecules closer to the target values than the others. In addition, MoGAT achieved the lowest RMSE of 0.4784. Thus, we demonstrated that our method outperformed the other baseline methods in solubility prediction. In other words, we experimentally showed that the performance is improved by using the information transmitted from various neighboring orders for final prediction as compared with AttentiveFP. Furthermore, as shown in Fig. 2b, we provide generalized error distribution (symmetric generalized Gaussian distribution)^32^ of the errors between the predicted and experimental solubility values. The generalized error distribution has three parameters, $\mu$, $\alpha$, and $\beta$. $\mu$ is the location parameter that determines the location or shift of the distribution^33^; $\alpha$ is the scale parameter that determines the dispersion, which means how spread out the errors are; $\beta$ is the shape parameter, which affects the shape of a distribution, such as peakedness, or fat-tailedness^34^. The estimated parameters of fitted generalized error distributions are provided in Table 3.

**Table 3:** Estimated parameters of generalized error distributions corresponding to the proposed and baseline methods.

	GCN	Weave	MPNN	AttentiveFP	MoGAT
$\mu$	0.118	− 0.260	0.089	0.056	− 0.007
$\alpha$	0.817	0.693	0.674	0.488	0.315
$\beta$	1.517	1.421	1.261	1.104	0.946

$\mu$, $\alpha$, and $\beta$ are location, scale, and shape parameters of the distribution, respectively.

As shown in Table 3, when comparing the parameters of the distributions, MoGAT and AttentiveFP, which are the variants of graph attention networks, had smaller $\mu$, $\alpha$, and $\beta$; the predictive performances of these graph attention-based methods show better solubility predictive performances than GCN, Weave, and MPNN. Moreover, MoGAT showed stable predictive performance because it was not biased to one side as $\mu$ was closer to zero than AttentiveFP. For $\alpha$ and $\beta$ values, which denote the existence of large errors, MoGAT showed the smallest values; hence, we also demonstrated that our method outperformed the other methods.

MoGAT also achieved better performance for predicting other molecular properties, including solvation-free energy and lipophilicity, than the other baseline methods. The detailed results for these experiments can be found in Supplementary Tables S1–S3.

Furthermore, to validate the interpretability of MoGAT, we derived the relative importance (attention scores) calculated by Eq. (8) and compared them with the attention scores of AttentiveFP. Note that the larger size of the attention score, the greater the importance of the corresponding chemical component to the final prediction.

**Figure 3:**
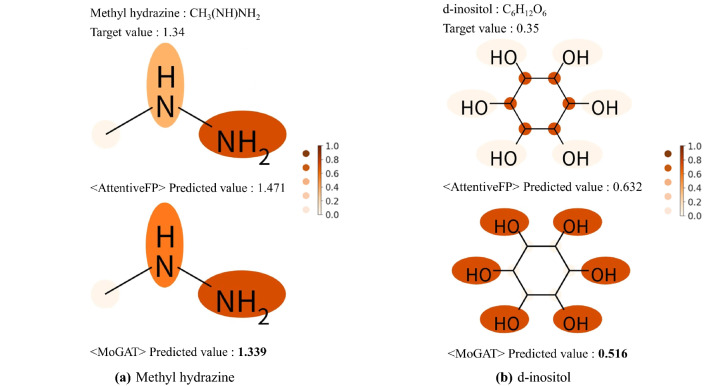
Relative importances of chemical components in computing water solubility predicted by AttentiveFP and MoGAT, for (**a**) methyl hydrazine and (**b**) d-inositol. The predicted water solubility values (unit: logS) are also provided for both AttentiveFP and MoGAT with experimentally measured values (target values). The attention scores are illustrated by the color-coding shown in the right panel.

Figure 3 shows the predicted water solubility and relative importance of each chemical component by the proposed MoGAT and AttentiveFP. The target value for water solubility was determined experimentally, as stated in the ESOL dataset. Figure 3a shows attention scores for water solubility of methyl hydrazine (CH$_3$(NH)NH$_2$) using AttentiveFP (top) and MoGAT (bottom). The experimental solubility value (target value) of methyl hydrazine was 1.34 (logS). MoGAT predicted water solubility to 1.339, resulting in a closer value to the true one than AttentiveFP. According to Klopman et al.^19^, which introduced a table of group contribution values of atoms to aqueous solubility, the contribution values of NH and NH$_2$ to water solubility were 0.9549 and 0.6935, respectively, while that of CH$_3$ was − 0.3361. In this case, both AttentiveFP and MoGAT emphasized the importance of NH and NH$_2$ over CH$_3$ as noted in Klopman et al.^19^. The attention scores in computing water solubility of d-inositol (C$_6$H$_{12}$O$_6$) using AttentiveFP (top) and MoGAT (bottom) are indicated in Fig. 3b. The target value of d-inositol was 0.35 (logS). AttentiveFP and MoGAT predicted water solubility as 0.632 and 0.516, respectively. Thus, we also confirmed that our method predicted the water solubility more accurately than AttentiveFP. In addition, the contribution value^19^ to the water solubility of OH was 1.0910, and that of C inside the ring system was − 0.4072. It is widely acknowledged that OH has a significant role in the water solubility of organic molecules with hydroxy groups^35^. Although MoGAT emphasized OH more than C, the attention scores derived by AttentiveFP differed from the known chemical fact as it predicted that C in the ring system is more important than OH in computing water solubility. The interpretability of MoGAT is still valid for other numerous molecules provided in Supplementary Figs. S1–S5.

Next, we examined how the predicted solubility values and attention scores change when some atoms (e.g., O and N) of the molecules are replaced with others (e.g., C).

**Figure 4:**
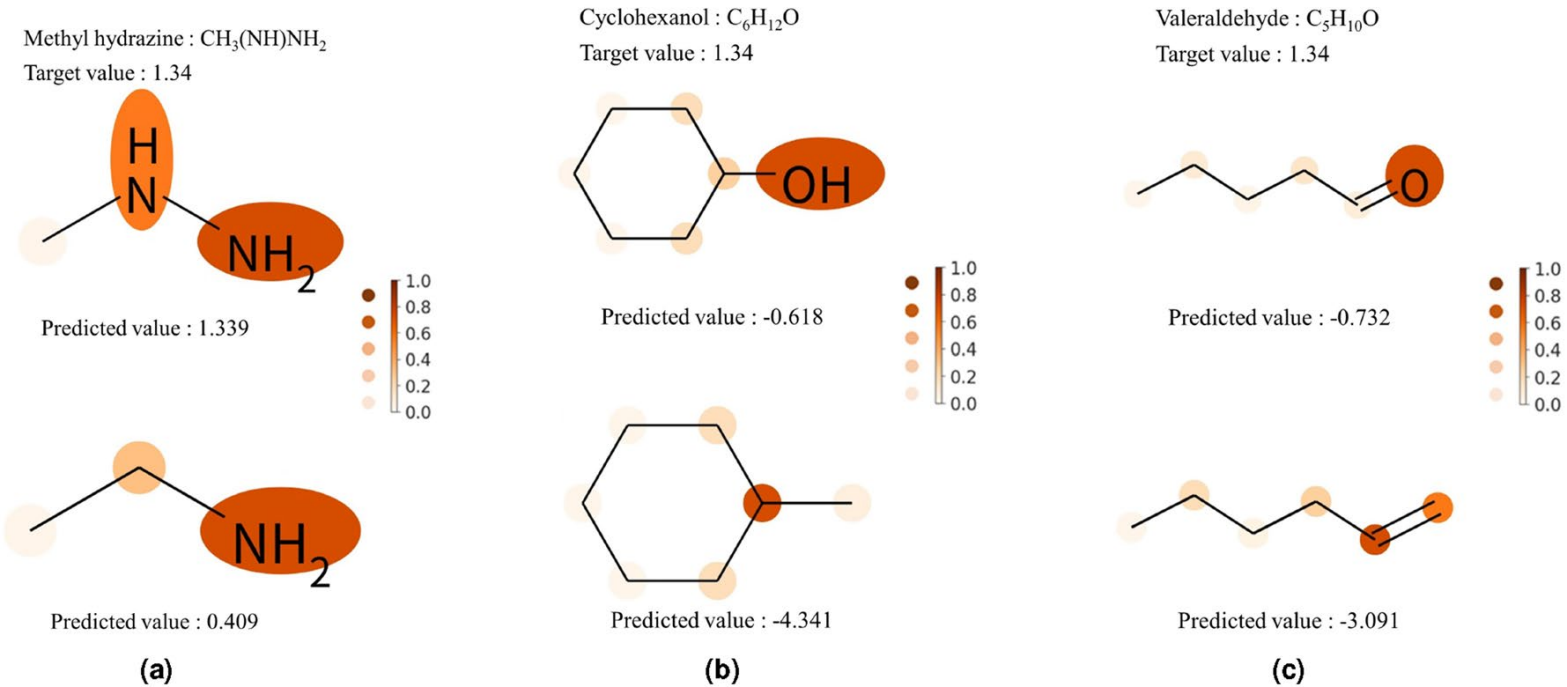
Change in water solubility and attention score estimated by MoGAT for some atomic or molecular replacements. The attention scores are presented by the color-coding shown in the right panel. (**a**) NH in methyl hydrazine is replaced with CH$_2$, (**b**) OH in cyclohexanol is replaced with CH$_3$, and (**c**) O in Valeraldehyde is replaced with CH$_2$. The unit of water solubility is logS.

In Fig. 4a, we changed NH in methyl hydrazine (CH$_3$(NH)NH$_2$) to CH$_2$. In the analysis of Klopman et al.^19^, the solubility contribution value of CH$_x$ is lower than those of OH, O, NH, and NH$_2$. Thus, in this case, we can observe the predicted solubility values were diminished than that of methyl hydrazine. Specifically, the target value of methyl hydrazine was 1.34 (logS), and the water solubility derived by MoGAT was 1.339 before the replacement, but after the replacement, it decreased to 0.409. In addition, the attention score of the replaced atom was also decreased. In Fig. 4b, OH in cyclohexanol (C$_6$H$_{12}$O) was replaced with CH$_3$, becoming methylcyclohexane (C$_7$H$_{14}$). The target values of cyclohexanol and methylcyclohexane were − 0.44 and − 3.85, respectively. In other words, when OH in cyclohexanol was replaced with CH$_3$, the water solubility is decreased. MoGAT predicted solubility values as − 0.618 and − 4.341, respectively, so the trend of predicted values is consistent with common knowledge. As in the previous case, the attention score of the replaced atom was also reduced when OH was replaced with CH$_3$. Finally, valeraldehyde (C$_5$H$_{10}$O) was compared to 1-hexene (C$_6$H$_{12}$), which is a form of valeraldehyde with the double bonded O replaced with CH$_2$, and the result was presented in Fig. 4c. The target values of valeraldehyde and 1-hexene are − 0.85 and − 3.23, respectively. The water solubility derived by MoGAT was decreased from − 0.732 to − 3.091 after the replacement and the attention score of the replaced atom was also lowered, which also implies the consistency of the results of MoGAT and the existing chemical knowledge. Other examples of the changes in water solubility when replacing atoms in various molecules are provided in Supplementary Figs. S6–S13.

### Commercial medicines

To verify the generalizability of MoGAT, we applied our method to the existing commercially available medicines, including rosuvastatin (C$_{22}$H$_{28}$FN$_3$O$_6$S), escitalopram (C$_{20}$H$_{21}$FN$_2$O), and ranolazine (C$_{24}$H$_{33}$N$_3$O$_4$). Rosuvastatin is a statin medicine used to prevent cardiovascular disease and treat dyslipidemia, escitalopram is an antidepressant for selective serotonin reabsorption inhibition, and ranolazine is a medicine used to treat heart-related chest pain^36^. Their solubility values were calculated by ALOGPS 2.1 in Tetko et al.^37^.

**Figure 5:**
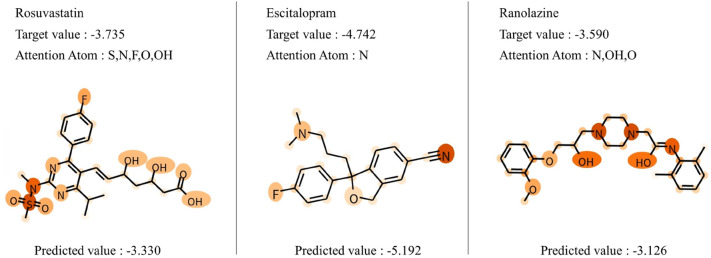
Predicted water solubility for three commercial medicines. The unit of water solubility is logS.

The water solubility values of the commercial medicines predicted by MoGAT are shown in Fig. 5. Given that the proposed MoGAT had RMSE of 0.4784 for the original test dataset (see Fig. 2a), the predictive performances for three commercial medicines not included in the ESOL dataset are also reliable, owing to predicted errors ranging from 0.4052 to 0.4635.

Moreover, when the atomic-specific attention scores were computed for these commercial medicines, nitrogen atoms had high attention scores in escitalopram and ranolazine, and sulfur and nitrogen atoms had high attention scores in rosuvastatin. According to Klopman et al.^19^, both the double-bonded sulfur and nitrogen have negative contributions of − 1.3197 and − 0.372, respectively, in a ring system. Since such negative contributions mean that they play important roles in low water solubility, the produced results by MoGAT were consistent with well-known chemical understandings, considering that a large molecule or polymer usually has low water solubility.

## Conclusion

In this paper, we proposed a novel graph attention neural network, MoGAT, for water solubility prediction to improve the predictive performance and interpret the predicted results. Specifically, we first generated graph embeddings for every node embedding layer; then, we created a final graph embedding using the graph embeddings and an attention mechanism. Generating graph embeddings from all node embedding layers can reflect various neighboring orders in the final prediction. Thus, MoGAT, which uses the final graph embedding combining all graph embeddings, improved predictive performance compared to the existing graph-based chemical property prediction methods. In addition, for atomic-specific importance to water solubility within a molecule, we demonstrated that the attention scores calculated by our method were consistent with existing chemical knowledge. Moreover, when specific chemical components of the molecule were replaced with others, the predicted solubility and atomic attention scores changed as expected based on the existing knowledge. However, our method has two limitations. First, since our method, MoGAT, computes graph embeddings from all node embedding layers and derives the final graph embedding by combining them, it has a higher time complexity than the existing methods. In terms of floating point operations (FLOPs), one of the indicators to measure the amount of calculation for a single instance in a model, MoGAT has 36.23 M FLOPs because of the additional parameters to generate the above-mentioned graph embedding layers, while AttentiveFP has 33.70 M FLOPs. Therefore, contriving an efficient approach, including refining the attention mechanism for obtaining graph embeddings, derived to handle this issue can be further studied. Second, the proposed method has several hyperparameters that should be optimized, including the number of node embedding and graph embedding updates. Thus, we can improve MoGAT by automatically finding the optimal hyperparameters or reducing them.

## Supplementary Information

Supplementary Information.

## Data Availability

All datasets used in this study are publicly available.
