# Supplementary material for: Multi-order graph attention network for water solubility prediction and interpretation
Source: Sci Rep. 2023 Mar 2;13:957. doi: 10.1038/s41598-022-25701-5 (PMC9981901; doi:10.1038/s41598-022-25701-5)
Supplement: Supplementary file 1 — Supplementary Information. [file 41598_2022_25701_MOESM1_ESM.pdf]

MoGAT: Multi-order Graph Attention Network for Water Solubility Prediction and Interpretation

SUPPLEMENTARY INFORMATION

Additional Experimental Results

Here, we report some additional experimental results to verify the effectiveness of MoGAT.

Other Molecular Properties Prediction

We first extended experiments with some benchmark datasets to validate the predictive performance of MoGAT for other molecular properties. The datasets included four regression tasks and seven classification tasks. The regression datasets consist of *Solubility*, *FreeSolv*, *Lipop*[1], and *QM9*[2]. The target of Solubility is water solubility, and those of FreeSolv and Lipop are solvation-free energy (kcal/mol) and lipophilicity (measured octanol/water distribution coefficient, logD), respectively. QM9 is a dataset for predicting 12 chemical properties of stable organic compounds consisting of hydrogen, carbon, oxygen, nitrogen, and fluorine. In specific, the targets of QM9 include dipole moment (DM, unit: Debye), isotropic polarization (IP, unit: $Bohr^3$), highest occupied molecular orbital (HOMO, unit: Hartree), lowest unoccupied molecular orbital (LUMO, unit: Hartree), band gap energy (BGE, unit: Hartree), electronic spatial range (ESR, unit: $Bohr^2$), zero vibration energy (ZVE, unit: Hartree), internal energies at 0K and 298.15K (IE, unit: Hartree), enthalpy (unit: Hartree), Gibbs free energy (GFE, unit: Hartree), and heat capacity (HC, unit: cal/mol$\times$K). The SMILES codes, which indicated molecular formulas and chemical structures as character strings, were given 130,000 instances in QM9 dataset, with the 12 target values for each data. In regression tasks, for comparison with AttentiveFP, we separated the QM9 dataset from the five regression datasets. For classification tasks, we used datasets related to bioactivity, physiology, and toxicity, including *MUV*, *HIV*, *BACE*, *Tox21*, *Toxcast*, *SIDER*, and *ClinTox*[1]. These tasks are binary classification, classifying whether a virus or toxic reaction exists.

Table S1. MAE on QM9 dataset for 12 chemical properties. The mean and standard deviation of repeated results are presented. For each target, the best performance is highlighted in boldface.

Type (unit)	GCN	Weave	MPNN	AttentiveFP	MoGAT
DM (Debye)	0.509 $\pm$ 8.0e-3	0.603 $\pm$ 3.9e-2	0.425 $\pm$ 4.7e-2	0.440 $\pm$ 1.1e-2	**0.417 $\pm$ 7.0e-3**
IP ($Bohr^3$)	0.746 $\pm$ 7.5e-2	1.198 $\pm$ 1.3e-1	0.675 $\pm$ 1.7e-1	0.468 $\pm$ 2.5e-2	**0.455 $\pm$ 2.6e-2**
HOMO (Hartree)	0.005 $\pm$ 2.4e-4	0.006 $\pm$ 2.8e-4	0.004 $\pm$ 8.5e-4	0.004 $\pm$ 3.0e-5	**0.003 $\pm$ 6.0e-5**
LUMO (Hartree)	0.005 $\pm$ 3.0e-5	0.007 $\pm$ 5.9e-4	0.005 $\pm$ 1.0e-3	0.004 $\pm$ 1.2e-4	**0.004 $\pm$ 1.0e-4**
BGE (Hartree)	0.007 $\pm$ 5.5e-4	0.008 $\pm$ 2.5e-4	0.006 $\pm$ 1.4e-3	0.005 $\pm$ 9.0e-5	0.005 $\pm$ 7.0e-5
ESR ($Bohr^2$)	46.511 $\pm$ 2.4e-0	50.684 $\pm$ 1.8e-0	29.517 $\pm$ 1.3e-0	28.363 $\pm$ 1.5e-0	**26.378 $\pm$ 1.1e-0**
ZVE (Hartree)	0.002 $\pm$ 6.2e-4	0.003 $\pm$ 1.3e-3	0.002 $\pm$ 4.9e-4	**0.001 $\pm$ 1.0e-5**	0.001 $\pm$ 1.0e-4
IE 0K (Hartree)	2.226 $\pm$ 2.4e-1	2.126 $\pm$ 3.7e-1	1.672 $\pm$ 4.9e-1	0.874 $\pm$ 2.5e-2	**0.794 $\pm$ 1.4e-1**
IE 298.15K (Hartree)	2.231 $\pm$ 2.5e-1	2.124 $\pm$ 3.8e-1	1.665 $\pm$ 4.9e-1	0.871 $\pm$ 2.2e-2	**0.794 $\pm$ 1.4e-1**
Enthalpy (Hartree)	2.230 $\pm$ 2.5e-1	2.118 $\pm$ 3.8e-1	1.658 $\pm$ 4.8e-1	0.871 $\pm$ 2.2e-2	**0.794 $\pm$ 1.4e-1**
GFE (Hartree)	2.230 $\pm$ 2.4e-1	2.121 $\pm$ 3.8e-1	1.663 $\pm$ 4.8e-1	0.871 $\pm$ 2.2e-2	**0.794 $\pm$ 1.4e-1**
HC (cal/mol·K)	0.338 $\pm$ 8.0e-3	0.529 $\pm$ 6.5e-2	0.310 $\pm$ 8.6e-2	**0.235 $\pm$ 1.8e-2**	0.240 $\pm$ 8.0e-3

Tables S1 and S2 list mean absolute error (MAE) of the QM9 and RMSE of the rest regression tasks, respectively. In both the QM9 and the rest datasets, MoGAT showed a better prediction than the other existing methods.

Table S3 indicates AUROC scores for the classification tasks. MoGAT showed superior performance compared to the other baseline methods in five out of seven classification tasks.

Water Solubility Prediction

Next, for various molecules, we compared AttentiveFP and MoGAT in terms of the importance scores for each chemical component in a molecule and predicted water solubility values. In Figures S1-S5, the component symbol of carbon and hydrogen connected to carbon are omitted; the names and chemical formulas of the molecules are indicated at the top, and the target values are presented bottom; the results of MoGAT is shown on the left, and that of AttentiveFP on the right.

Table S2. RMSE on the rest regression tasks except for QM9. For each dataset, the best performance is highlighted in boldface.

Type	Solubility (unit: logS)	Freesolv (unit: kcal/mol)	Lipop (unit: logD)
GCN	0.723	1.130	0.990
Weave	0.460	1.304	1.125
MPNN	0.350	1.113	0.913
AttentiveFP	0.286	0.920	0.617
MoGAT	**0.281**	**0.835**	**0.599**

Table S3. AUROC on classification tasks. This score indicates that the closer to 1, the better the prediction performance. The mean and standard deviation of repeated results are presented. For each dataset, the best performance is highlighted in boldface.

Type	HIV	BACE	MUV	Toxcast	Tox21	SIDER	ClinTox
GCN	0.788 ± 0.011	0.863 ± 0.011	0.709 ± 0.062	0.710 ± 0.003	0.819 ± 0.004	0.592 ± 0.015	0.841 ± 0.020
Weave	0.501 ± 0.002	0.755 ± 0.012	0.500 ± 0.002	0.723 ± 0.004	0.793 ± 0.002	0.555 ± 0.019	0.852 ± 0.031
MPNN	0.742 ± 0.028	0.869 ± 0.009	0.485 ± 0.021	0.733 ± 0.004	0.816 ± 0.006	0.585 ± 0.029	0.848 ± 0.026
Attentive FP	0.795 ± 0.026	**0.874** ± **0.012**	**0.806** ± **0.027**	0.835 ± 0.006	0.844 ± 0.009	0.620 ± 0.017	0.943 ± 0.007
MoGAT	**0.821** ± **0.003**	0.873 ± 0.015	0.804 ± 0.016	**0.851** ± **0.006**	**0.846** ± **0.012**	**0.641** ± **0.015**	**0.964** ± **0.012**

The predictive performances for some molecules of the proposed MoGAT achieved slightly lower than those of AttentiveFP. However, MoGAT provided more reasonable importance scores than AttentiveFP.

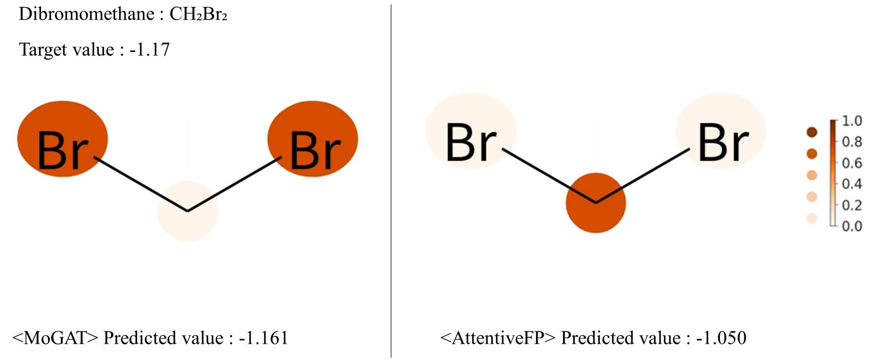

Figure S1. The predicted water solubility (unit: logS) and importance of each atom for dibromomethane molecule. The attention scores are presented by the color coding in the right panel.

Replacement of Some Components in Molecules

In this section, for various molecules, we confirmed how the predicted values and attention scores changed when some chemical components of the molecules were replaced with others. For example, O, OH_x, and NH_x are replaced with CH_x, or CH_x was substituted with O or OH_x. In Figures S6-S13, the component symbol of carbon and hydrogen connected to carbon were omitted. In addition, the names and chemical formulas of the molecules are indicated at the top, and the target values are presented bottom. Finally, the substituted atoms and chemical formulas before and after transformation were shown for each molecule.

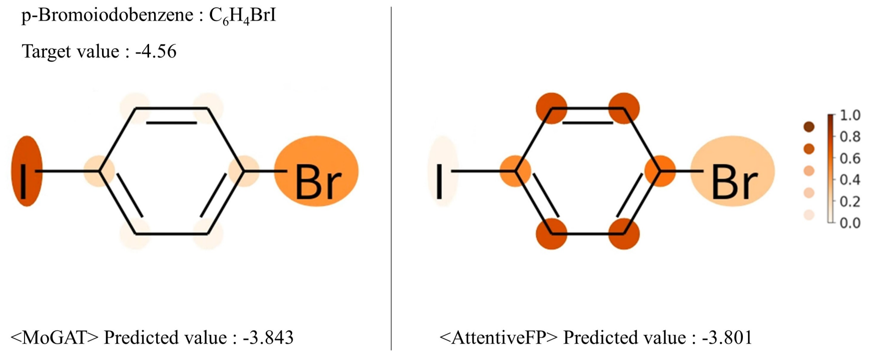

Figure S2. The predicted water solubility (unit: logS) and importance of each atom for p-bromoiodobenzene molecule. The attention scores are presented by the color coding in the right panel.

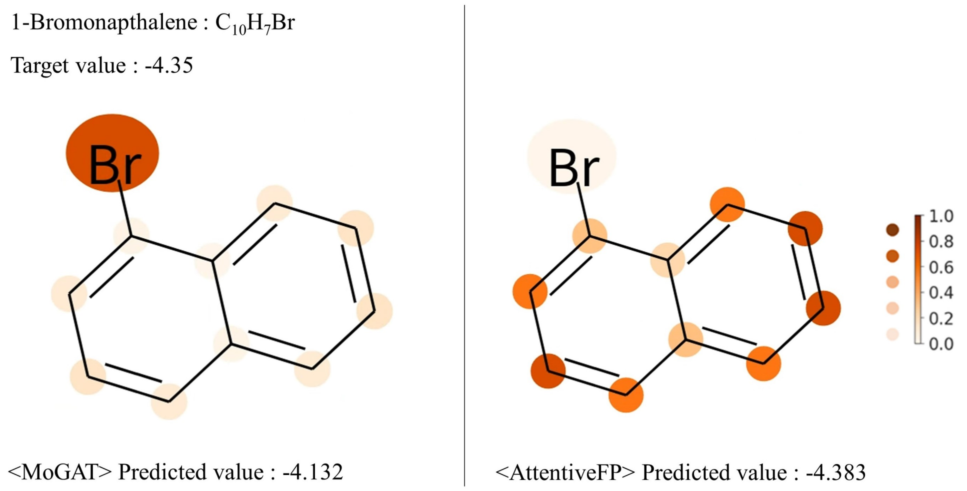

Figure S3. The predicted water solubility (unit: logS) and importance of each atom for 1-bromonapthalene molecule. The attention scores are presented by the color coding in the right panel. In this case, the proposed method had a slightly larger prediction error than AttentiveFP; however, it shows better attention results than AttentiveFP.

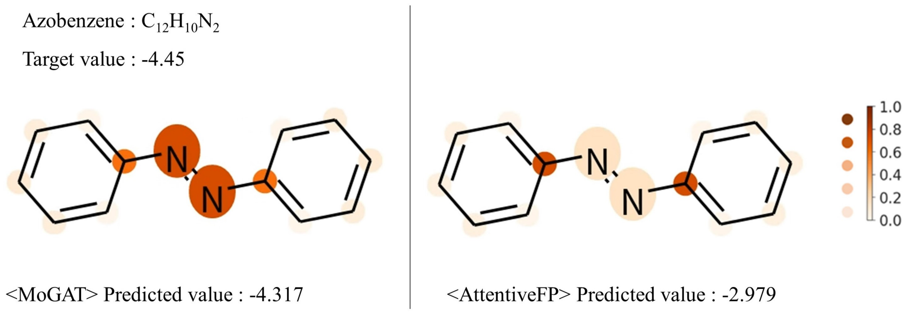

Figure S4. The predicted water solubility (unit: logS) and importance of each atom for azobenzene molecule. The attention scores are presented by the color coding in the right panel.

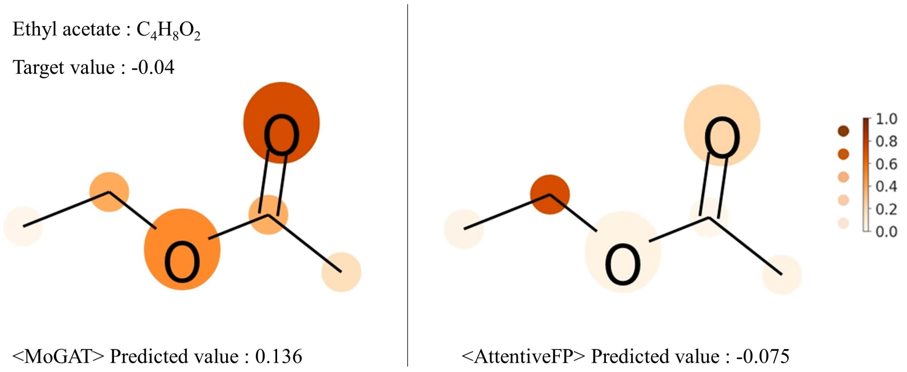

Figure S5. The predicted water solubility (unit: logS) and importance of each atom for ethyl acetate molecule. The attention scores are presented by the color coding in the right panel. In this case, the proposed method had a slightly larger prediction error than AttentiveFP; however, it shows better attention results than AttentiveFP.

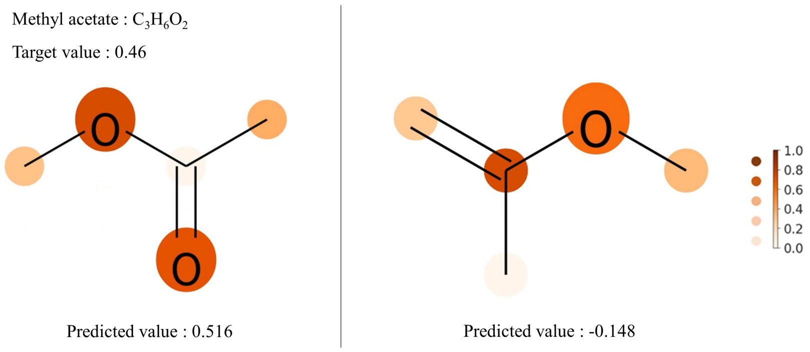

Figure S6. Change in water solubility and attention scores when one of oxygen atoms in methyl acetate is substituted with $-CH_2$. The unit of water solubility is logS and attention scores are presented by the color coding in the right panel.

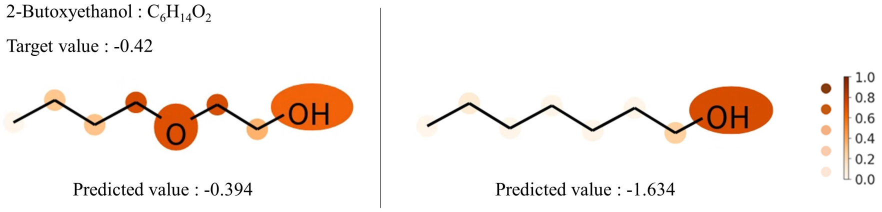

Figure S7. Change in water solubility and attention scores when one of oxygen atoms in 2-butoxyethanol is substituted with $-CH_2$. The unit of water solubility is logS and attention scores are presented by the color coding in the right panel.

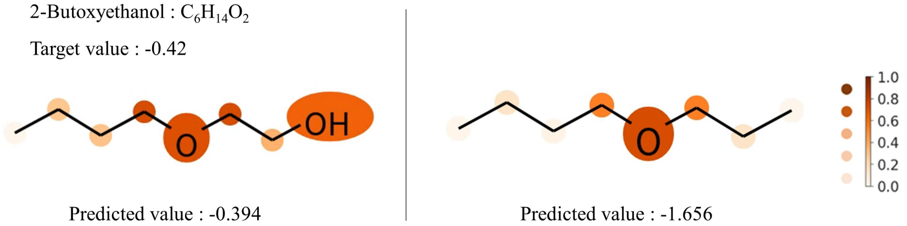

Figure S8. Change in water solubility and attention scores when an -OH in 2-butoxyethanol is substituted with $-CH_3$. The unit of water solubility is logS and attention scores are presented by the color coding in the right panel.

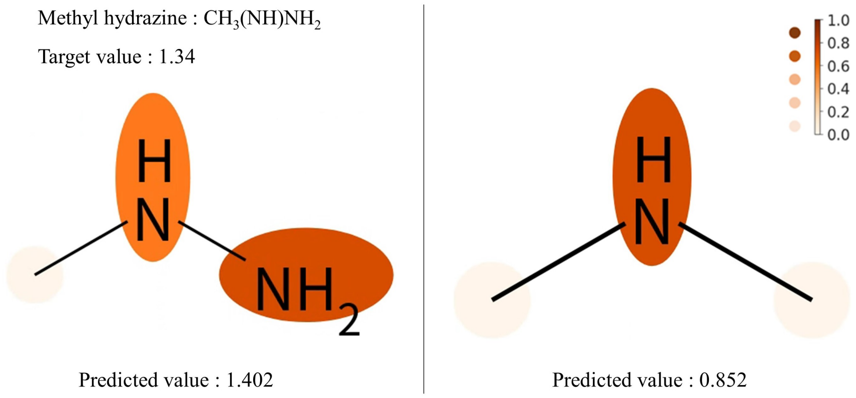

Figure S9. Change in water solubility and attention scores when a -NH2 in methyl hydrazine is substituted with $-CH_3$. The unit of water solubility is logS and attention scores are presented by the color coding in the right panel.

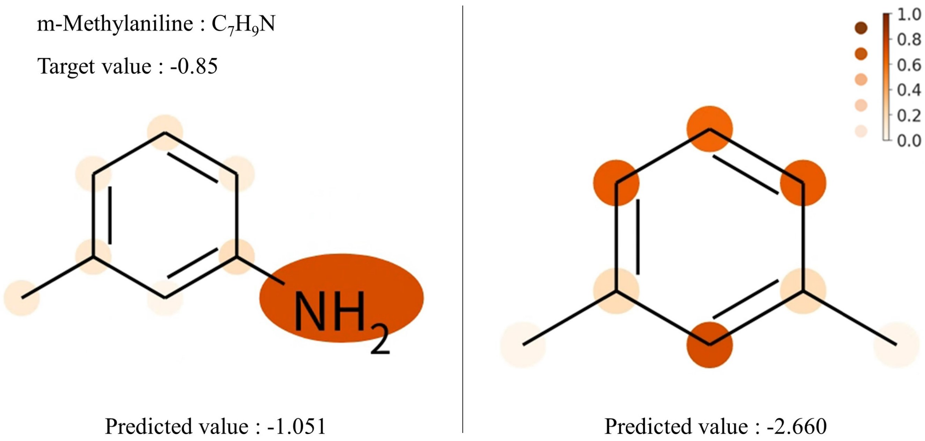

Figure S10. Change in water solubility and attention scores when a -NH2 in m-methylaniline is substituted with $-CH_3$. The unit of water solubility is logS and attention scores are presented by the color coding in the right panel.

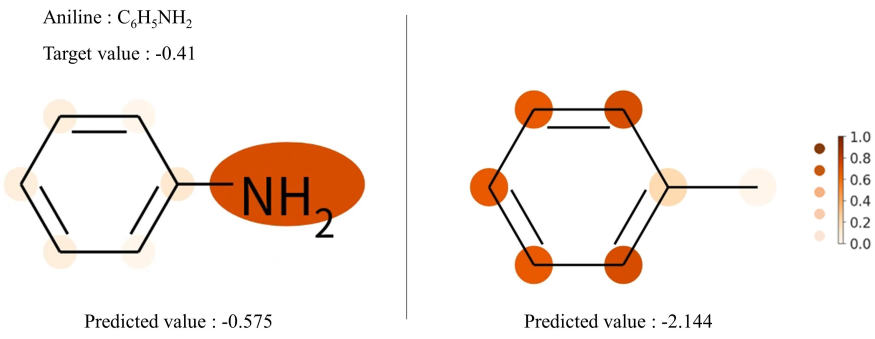

Figure S11. Change in water solubility and attention scores when a -NH2 in aniline is substituted with $-CH_3$. The unit of water solubility is logS and attention scores are presented by the color coding in the right panel.

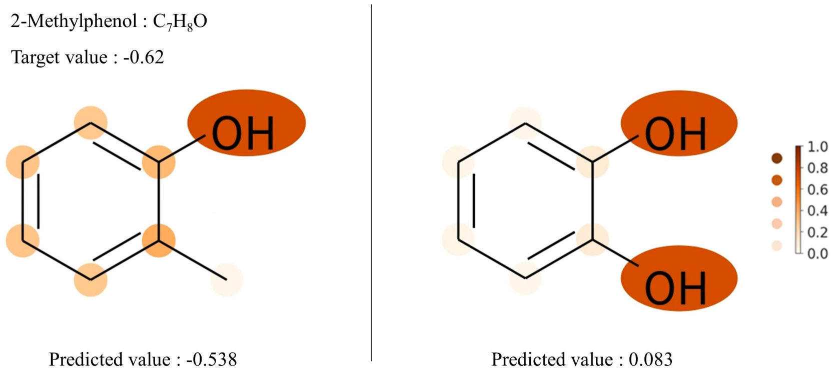

Figure S12. Change in water solubility and attention scores when a -CH_3 in 2-methylphenol is substituted with an -OH. The unit of water solubility is logS and attention scores are presented by the color coding in the right panel.

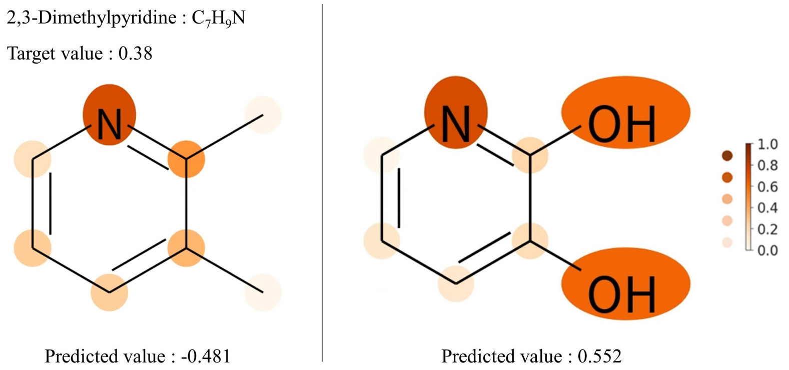

Figure S13. Change in water solubility and attention scores when -CH_3 in 2,3-dimethylpyridine are substituted with two -OHs. The unit of water solubility is logS and attention scores are presented by the color coding in the right panel.

References

1. Wu, Z. *et al.* Moleculenet: a benchmark for molecular machine learning. *Chem. science* **9**, 513–530 (2018).

2. Ruddigkeit, L., Van Deursen, R., Blum, L. C. & Reymond, J.-L. Enumeration of 166 billion organic small molecules in the chemical universe database gdb-17. *J. chemical information modeling* **52**, 2864–2875 (2012).
